# Dimorphic flowers modify the visitation order of pollinators from male to female flowers

**DOI:** 10.1038/s41598-020-66525-5

**Published:** 2020-06-19

**Authors:** Kaoru Tsuji, Kazuya Kobayashi, Eisuke Hasegawa, Jin Yoshimura

**Affiliations:** 1 Center of Ecological Research, Kyoto University, Otsu, Shiga 520-2113 Japan; 2 Hokkaido Forest Research Station, Field Science Education and Research Center, Kyoto University, 553 Tawa, Shibecha-cho, Kawakami-gun, Hokkaido 088-2339 Japan; 3 Laboratory of Animal Ecology, Department of Ecology and Systematics, Graduate School of Agriculture, Hokkaido University, Hokkaido, 060-8589 Japan; 4 Department of Mathematical and Systems Engineering, Shizuoka University, Hamamatsu, Japan; 5 Department of International Health, Institute of Tropical Medicine, Nagasaki University, Nagasaki, 852-8523 Japan; 6 Department of Environmental and Forest Biology, State University of New York College of Environmental Science and Forestry, Syracuse, New York USA; 7 Marine Biosystems Research Center, Chiba University, Kamogawa, Chiba Japan; 8 Department of Biological Sciences, Tokyo Metropolitan University, Hachioji, Tokyo 192-0397 Japan; 9 The University Museum, University of Tokyo, Bunkyo-ku, Tokyo 113-0033 Japan

**Keywords:** Ecology, Evolution

## Abstract

Sexual dimorphism is a pervasive form of variation within species. Understanding how and why sexual dimorphism evolves would contribute to elucidating the mechanisms underlying the diversification of traits. In flowering plants, pollinators are considered a driver of sexual dimorphism when they affect female and male plant fitness in distinct ways. Here, we found that flowers appear to manipulate the behavior of pollinators using sexually dimorphic traits in the dioecious tree *Eurya japonica*. In this plant, female flowers present a higher-quality reward for pollinators, whereas male flowers have a more conspicuous appearance. Plants benefit by inducing pollinators to carry pollen from male to female flowers, and their sexual dimorphism might thus facilitate pollen movement through pollinator behavior. In two-choice experiments, pollinators frequently moved from male to female flowers, whereas computer simulation suggested that sexually dimorphic traits would evolve if pollinators changed behavior depending on the traits of the flowers they had just visited. These results suggest that the floral traits affecting the visiting order of pollinators have evolved in plants. Using *E. japonica*, we theoretically show that the induction of sequential behavior in pollinators might be crucial to the evolution of sexual dimorphism in flowers, and our experiments support these findings.

## Introduction

Sexual dimorphism is a pervasive form of intraspecific variation, and flowers, the reproductive organs of plants, exhibit various sexually dimorphic traits^1–8^. Plants often require vectors of pollen transfer such as animal pollinators. Flower-pollinator interactions can therefore theoretically drive the evolution of plant sexual dimorphism^2–7^. A well-established hypothesis on how sexual dimorphism in floral traits arises from flower-pollinator interactions is that of sexual selection, proposed by Bell^1^. In insect-pollinated species, plants with larger male flowers, such as *Salix caprea*^9^ and *Wurmbea dioica*^10^, are more common than plants with larger female flowers^11^. Here we regard plants with larger male flowers as exemplars of how pollinators are related with flower sexual dimorphism. Bell’s hypothesis of sexual selection on male flowers assumes that male reproductive success increases linearly with the number of pollinators, whereas female reproductive success is limited by resource availability^12^. In other words, male flowers benefit from additional pollinators more so than do female flowers, and plant species with larger male flowers than female flowers are considered examples supporting this hypothesis^3,9,13^ because larger flowers attract more pollinators^1,14–16^. This explanation derives from Bateman’s principle^17^ by focusing on the differences in strategies for maximizing male or female fitness^1,3^. This hypothesis has been theoretically supported^12,18^ and applied in some cases (e.g., nectar, scent, and size), including a set of traits such as larger male flowers with sweeter nectar^9,13,19^.

Nevertheless, sexual dimorphism in flowers can be explained by another hypothesis. Müller^20^ proposed a verbal model in which variations in flower size have evolved to guide pollinators from larger to smaller flowers. Although the applicability of this model might depend on pollinator species, this sequential foraging of pollinators could improve the pollination efficiency of individual plants in both sexes. In other words, flowers appear to manipulate pollinators via sexual dimorphism to maximize the fitness of both male and female trees (hereafter “sequence hypothesis”). Compared to Bell’s hypothesis on sexual selection, the sequence hypothesis has rarely been examined to date, although Abraham^21^ showed that size differences in althea flowers mediate the visiting sequence of native free-flying bumblebees, i.e., they visit larger flowers first and then smaller flowers. The sequence hypothesis could play a critical role in dioecious animal-pollinated plants because dioecious plants have sexually separate individuals and the plants require pollinators to carry pollen from males to females. Nonetheless, to the best of our knowledge, the sequence hypothesis has not been fully developed theoretically nor tested.

Bell’s hypothesis and the sequence hypothesis are not mutually exclusive, and both can be applicable in plants in which male flowers are larger than female flowers, but provide contrasting predictions regarding reward traits (e.g., nectar amount or quality). Bell’s hypothesis predicts that male flowers provide a richer reward for pollinators (e.g., greater quantity, higher quality of nectar, or both). In contrast, the sequence hypothesis predicts that male flowers would provide less reward because the combination of a stronger signal from male flowers and the higher reward from female flowers encourages pollinators to move from male to female plants. That is, Bell’s hypothesis in insufficient to explain the small reward offered by larger male flowers, but it can be explained by the sequence hypothesis. However, few studies on the sequence hypothesis have considered both advertisement traits and reward traits simultaneously^5,21^, which makes it difficult to evaluate which of the two hypotheses is more likely to explain sexual dimorphism in floral traits.

**Figure 1:**
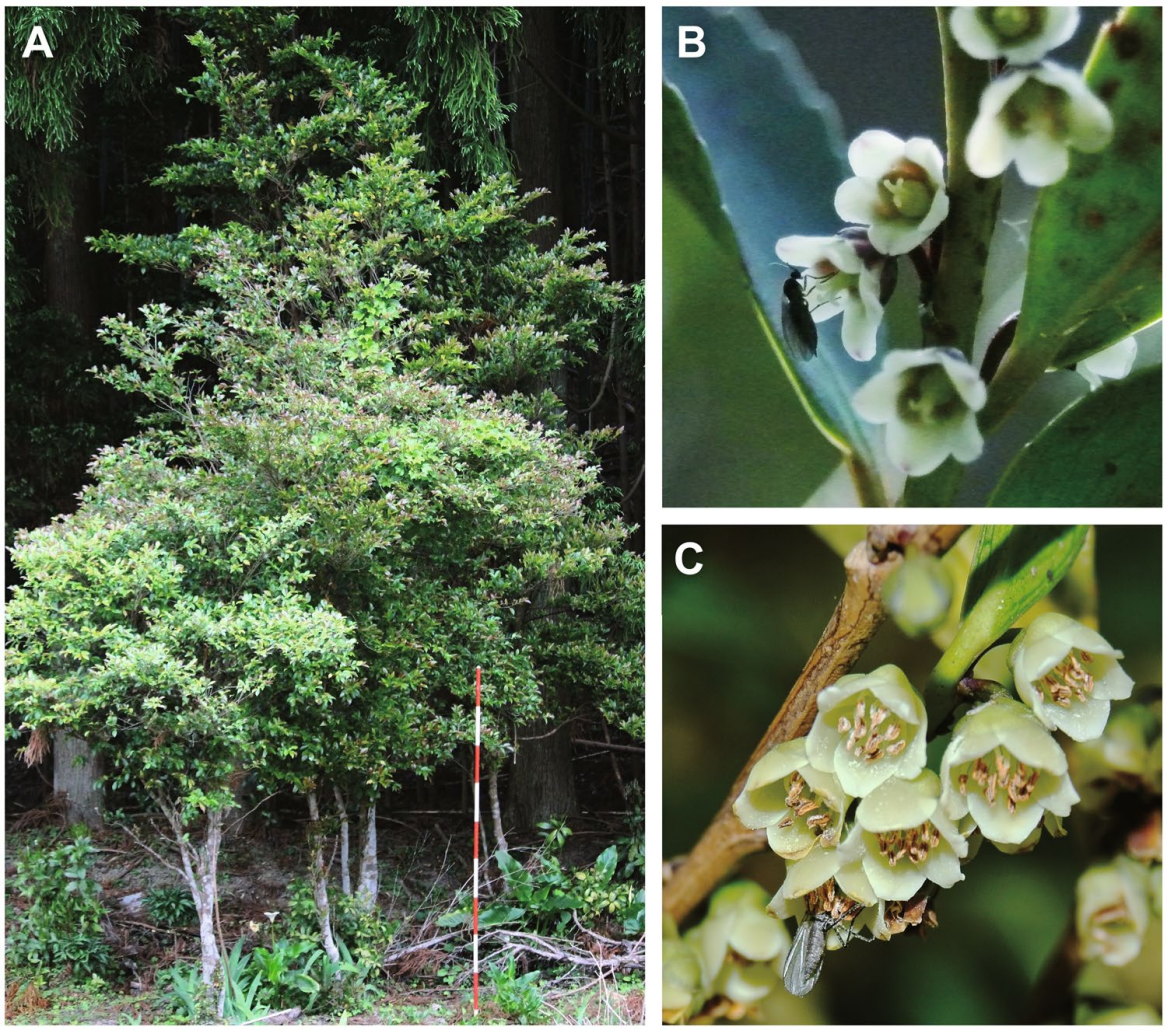
Plant material, *Eurya japonica* trees (photographs: K. Tsuji). Male and female flowers are borne on different plants and not on different twigs of the same plant. (**A**) Photo of trees with a 2-m surveying pole (the red and white sections indicate 20 cm). (**B**) Female flowers. (**C**) Male flowers.

**Figure 2:**
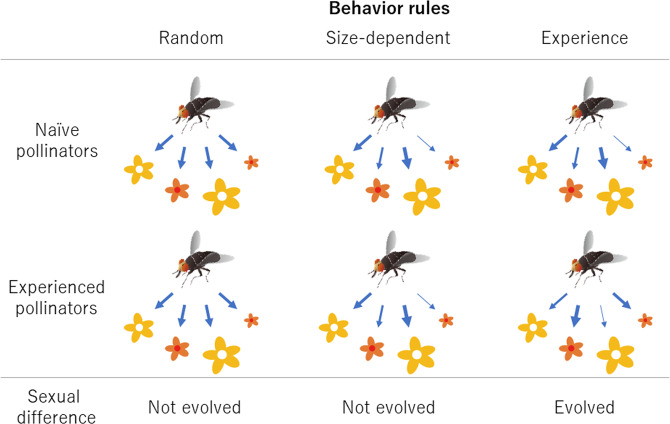
Conceptual diagram showing whether the behavioral rules when pollinators choose flowers to be visited leads to sexual dimorphism. In the simulations, each flower has a different size and nectar amount. As an example, we illustrate above yellow and orange flowers to show relatively large flowers without nectar, and relatively small flowers with nectar, respectively (yellow and orange do not show differences in floral color). Red in the center of orange flowers shows nectar reward and open circles in the center of yellow flowers show no nectar reward. Flowers and flies are drawn by K. Tsuji and Y. Kanzaki, respectively. The left: random model assumes that pollinators decide flowers randomly without recognition of flower size and nectar amount. Under the no selective pressure on floral size and nectar amount, no evolution of sexual difference is predicted. The middle: size-dependent model assumes that pollinators decide flowers based on floral size. In this model, since female-female and male-male competitions occur, large flowers are evolved, but sexual difference is not evolved. The right: experience model assumes that naïve pollinators will decide flowers based on flower size, and experienced pollinators choose flowers based on the nectar reward of their previous visited flower. For example, if a naïve pollinator visits the largest flower without nectar at first, the pollinator next chooses flower with a different size (i.e., smaller flower than the largest one) and avoids visiting such large flower without nectar. This pollinator behavior drives the evolution of sexual differences in floral traits: in male flowers, large flowers are evolved to attract naïve pollinators and less nectar reward to make experienced pollinators with pollen avoid the flowers with the same sizes as the male itself, while in female flowers, flowers with different size from male flowers are evolved to attract pollinators that has already visited nectar less male flowers.

In this study, we focused on both advertisement and reward traits. As a model system, we used the dioecious tree *Eurya japonica* (Thunb). This tree grows more than 10 m height and 20 cm diameter at breast height (Fig. 1A) and is pollinated not by wind, but by insects including flies, midges, bees, wasps, beetles, butterflies and moths^22^ (Fig. 1B,C). In this plant species, male flowers are more showly (i.e., are larger) than female flowers, whereas female flowers produce an enhanced reward (i.e., nectar with more sugar) compared to male flowers (Table 1). To test whether the sequence hypothesis can be empirically tested with this tree species (i.e., whether pollinators moved from male flowers to female flowers), we performed laboratory experiments to evaluate the behavior of pollinators on female and male flowers. We also tested whether the sequence hypothesis can theoretically explain the evolution of the sexual dimorphism: male flowers invest in advertisements; females, in rewards. We conducted simulations based on two mathematical models in which we hypothesized that the pollinators decide which flowers to visit next based only on the flower size (size-dependent model) or both the flower size and reward (experience model) (see Fig. 2).

**Table 1:** .Petal length, the amount and concentration of sugar in nectar, and the number of flowers per cm of twig on female and male *Eurya japonica* plants.

	Female plants (average± SE)	Male plants (average± SE)	Data source
Perianth length***	3.2 ± 0.0 mm	4.9 ± 0.0 mm	Tsuji & Ohgushi^22^
Total sugar amount per flower**	0.34 ± 0.02 μmol	0.20 ± 0.01μmol	Tsuji & Fukami^32^
Total sugar concentration*	0.08 ± 0.01 mol/L	0.07 ± 0.01 mol/L	Tsuji & Fukami^32^
Sucrose amount per flower**	0.25 ± 0.01 μmol	0.14 ± 0.00 μmol	Tsuji & Fukami^32^
Glucose amount per flower**	0.04 ± 0.00 μmol	0.03 ± 0.00 μmol	Tsuji & Fukami^32^
Fructose amount per flower	0.05 ± 0.00 μmol	0.04 ± 0.00 μmol	Tsuji & Fukami^32^
Sucrose concentration**	0.05 ± 0.00 mol/L	0.04 ± 0.00 mol/L	Tsuji & Fukami^32^
Glucose concentration	0.01 ± 0.00 mol/L	0.01 ± 0.00 mol/L	Tsuji & Fukami^32^
Fructose concentration	0.01 ± 0.00 mol/L	0.02 ± 0.00 mol/L	Tsuji & Fukami^32^
Flower number per 1 cm twigs	8.8 ± 0.1	8.4 ± 0.1	Table S1
Flower number per 1 cm twigs			
Plant pair ID in the experiment			
1	14.8 ± 0.6	13.1 ± 0.9	Table S1
2	8.1 ± 0.3	8.9 ± 0.2	Table S1
3	7.0 ± 0.5	6.8 ± 0.4	Table S1
4	7.9 ± 0.6	7.7 ± 0.4	Table S1
5	10.1 ± 1.6	7.5 ± 1.4	Table S1
6	7.9 ± 0.3	7.9 ± 0.9	Table S1
7	10.5	7.2	Table S1
8	9.1	6.5	Table S1
9	6.6 ± 0.7	6.5 ± 0.8	Table S1
10	6.8 ± 0.5	3.8 ± 0.8	Table S1

*<0.05, **<0.01, ***<0.001.

See Table S3 for statistics for perianth length, sugar amount, and flower number. Statistics for sugar concentration was published in Tsuji & Fukami^32^.

## Results

**Figure 3:**
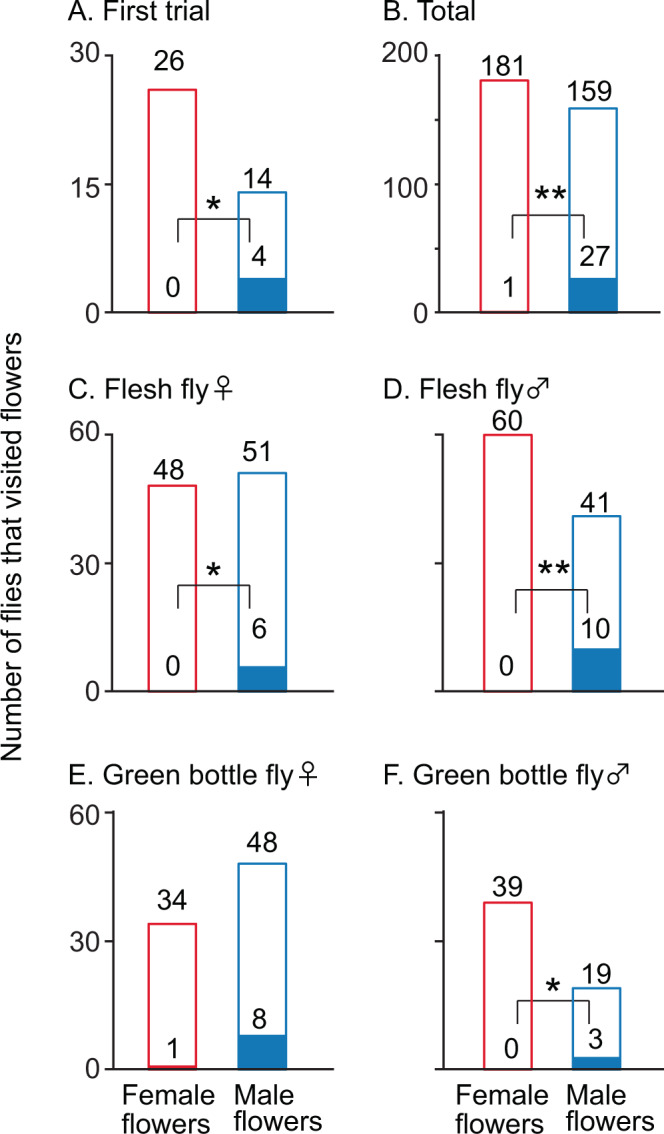
Movement of flies from male to female flowers. The solid-filled bars show the number of flies that moved from female or male twigs to a twig of the opposite sex. The open-filled bars show the number of flies that stayed on the twigs of one sex for 1 minute. (**A**) First trial for each twig pair in a day. (**B**) Total of the trial. (**C**) Female flesh flies. (**D**) Male flesh flies. (**E**) Female green bottle flies. (**F**) Female green bottle flies. No bias in the first choice was observed in (**A**–**E**), but female bias was observed in (**F**) (binomial tests, *P* = 0.08, 0.3, 0.9, 0.07, 0.2, and 0.01, respectively). Flies more frequently moved from male flowers to female flowers (chi-squared test: *P* = 0.01, <0.01, 0.03, <0.01, 0.15 and 0.04, respectively). ** and * show *P* < 0.01 and 0.05, respectively.

**Figure 4:**
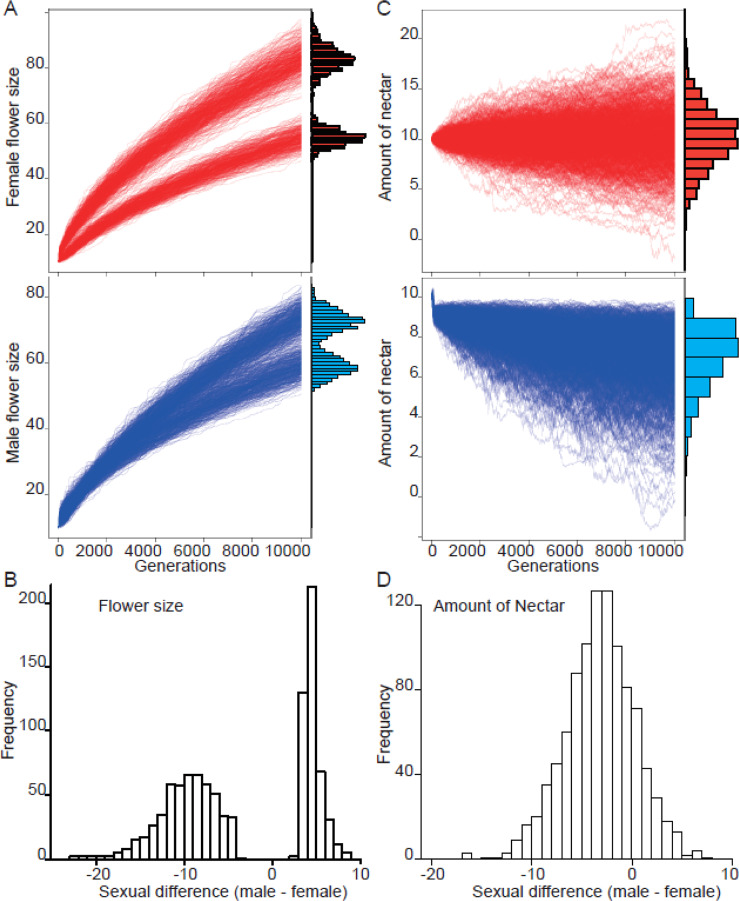
Simulation results obtained for pollinators using the experience rule. (**A**) Evolutionary dynamics of female and male flower size. Each line represents a single simulation result. The distribution of flower size at the end of the simulations is shown as a histogram on the right side. (**B**) Sexual differences in flower size observed at the end of the simulations. (**C**) Evolutionary dynamics of the amount of nectar in female and male flowers. Each line represents a single simulation result. The distribution of the nectar amounts at the end of the simulations is shown as a histogram on the right side. (**D**) Sexual differences in the amount of nectar observed at the end of the simulations.

In the empirical two-choice experiment, the frequency of pollinator movement from male to female flowers is significantly higher than the opposite movement (i.e., from female to male flowers) (chi-squared test^23^: P < 0.01, see details in Fig. 3A,B, Table S1). Given their first choice, flies visited female and male flowers equally (Fig. 3A,B). This tendency was observed with two different fly species, even though the sex or species of the flies might affect their foraging (Fig. 3C–F, Table S1). In the simulations with the random rule for pollinators, the flower size (Fig. S1A) and nectar amount (Fig. S1C) randomly evolved in both sexes, and the sexual differences in flower size (Fig. S1B) and nectar amount (Fig. S1D) showed a unimodal distribution around zero. In the size-dependent model, although large flowers evolved in both sexes (Fig. S2A), sexual dimorphism did not evolve in terms of flower size (Fig. S2B) or nectar amount (Fig. S2C,D). In contrast, the simulations exploring whether the pollinators decided which flower to visit next depending on flower size and their experience showed not only the evolution of larger flowers but also bimodal distributions for flower size in both sexes at the end of the simulations (Fig. 4A). These bimodal distributions reflected the evolution of sexual dimorphism, as confirmed by the fact that the sexual difference in flower size at the end of the simulations showed a bimodal distribution avoiding a neighbor of zero (Fig. 4B). Note that in some cases, male flowers evolved to exhibit a larger size than female flowers, and female flowers evolved to exhibit a larger size than male flowers (larger male flowers than female flowers occurred in 512 out of 1,000 simulation results; binomial test *P* = 0.467). Moreover, the amount of nectar in male flowers rapidly declined to below the threshold (10), which caused the pollinators to ignore flower size when making decisions about flower visitation. This was not observed in female flowers (Fig. 4C). As a result, the amount of nectar in female flowers was higher than in male flowers (Fig. 4D); 818 of 1,000 simulations showed that nectar production in female flowers was greater than that in male flowers; binomial test *P* < 0.001).

**Figure 5:**
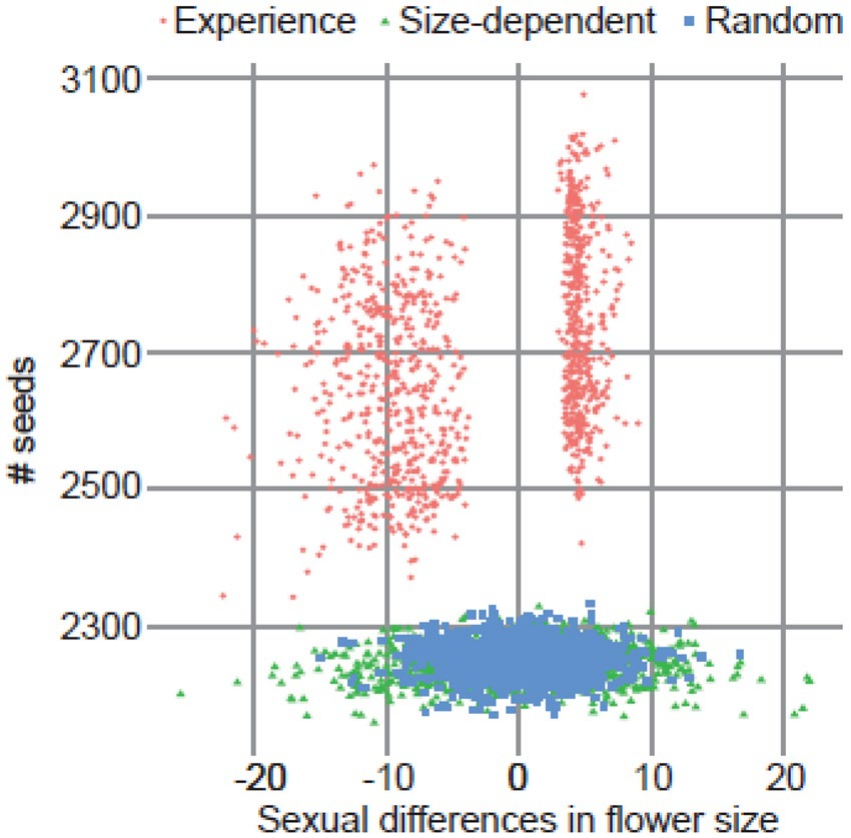
Effect of sexual dimorphism on pollination success rates. Each point represents a single simulation result. The red circles, green triangles and blue squares correspond to the simulations assuming the “experience”, “size-dependent” and “random” rules, respectively.

We also compared the number of successful pollinations during the last generation of the simulations among all three rules (Fig. 5). When the pollinators used their experience to make decisions, more successful pollinations occur compared to the other rules used (ANOVA, total of 3000 simulation runs, effect of behavior rule d.f. = 2, *F* = 8646.5, *P* < 0.001; Tukey HSD, between “experience” and “random” *P* < 0.001, between “experience” and “size-dependent” *P* < 0.001, between “size-dependent” and “random” *P* = 0.379). Moreover, focusing only on the results of the experience rule, the number of successful pollinations was greater when males had larger flowers than females, compared to when male flowers were smaller (Fig. 5; *t* test, *t* = −13.253, d.f. = 966.72, *P* < 0.001). Mean seed production in the last generation was 2752.020 for large male flowers and 2638.609 for large female flowers.

## Discussion

The simulation results show that large male flowers with little reward are beneficial for male plants if the pollinator behaviors reflect their experience (Fig. 4) and if sexual dimorphism is beneficial for plant fitness (Fig. 5). The results suggest that pollinator experience can result in the evolution of sexual dimorphism in flowers. Although the experiment performed under artificial conditions in the small box did not completely support the sequence hypothesis, the results partially supported the hypothesis by showing a high frequency of movement from male to female flowers (Fig. 3). In other words, the size of the experimental box may have limited the ability of pollinator flies to appropriately recognize the floral traits of the tree species *E. japonica*. In this experiment, we could not control the floral scent, but the stronger scent of male flowers compared with female flowers^24^ suggests that floral scent might serve as an advertisement trait for pollinators in open air conditions. Prior experimental research has demonstrated partial support for the sequence hypothesis in another plant species, *Hibiscus syriacus L*., in which pollinators visit larger flowers first in the field^21^.

Pollinators can drive the evolution of flower traits^1,2,17,25,26^, larger flowers^1,14–16^, or flowers with rich rewards^27,28^, which attract more pollinators, as shown in *Chrysanthemum leucanthemum*^1^ and *Petunia axillaris*^29^. As predicted under the sexual selection hypothesis based on Bateman’s principle, larger male flowers, with richer rewards than female flowers, are observed^3,9,13^. In contrast to these examples, a few recent studies^22,30–32^ identified larger male flowers with fewer rewards than female flowers. This puzzling inconsistency in dimorphic flower traits requires the proposal of new evolutionary mechanisms, we propose that the induction of sequential behavior in pollinators is crucial to this inconsistency. More detailed empirical studies, however, are required to validate the sequence hypothesis because some other limiting factors such as floral herbivory or trade-off between flower size and other traits might act on small female flowers^4,13,33,34^.

The simulation results can also explain consistent sexually dimorphic traits (e.g., larger female flowers with richer rewards than male flowers^4^). When we incorporation the assumption of the experience rule in our simulations, approximately half of them resulted in the evolution of smaller male flowers than female flowers (Fig. 4B). This finding suggests that the sequential hypothesis provides an explanation for why female flowers exhibit more conspicuous and attractive reward traits than male flowers, as observed in *Silene latifolia*^35^. Delph *et al*.^11^ showed that male plants have larger flowers than female plants in 70% of 66 insect-pollinated species. This proportion is higher than that found in this study (51.2% with the experience model), and the differences in these proportions can be explained by pollination efficiency, as simulated efficiency decreased with the evolution of inconspicuous and unattractive male flowers (Fig. 5). Thus, selection for pollination efficiency will result in the removal of such inconspicuous and unattractive males.

We selected and modeled plant species lacking sexual differences in the blooming season^32^, although male flowers bloom earlier than female flowers in some plant species^36,37^. In such cases, before females begin to bloom, males need to provide enough nectar to attract pollinators owing to male-male competition for pollinators. Thus, larger male flowers with a greater amount of nectar would be temporally observed in species with sexually distinct blooming seasons. On a shorter time scale, sexual differences in nectar production may occur over the course of a single day. Previous studies have shown that pollinators favor large male flowers in the early morning^21,38^, suggesting they are naïve at this time of day. If female flowers bloom later than male flowers within a given day, larger male flowers will produce more nectar in the morning because of male-male competition for pollinators, until the female flowers bloom. These more general conditions will be an interesting topic to explore in terms of explaining sexual dimorphism and the nectar production schedule of plants. Notably, the mechanism described in this study can potentially be applied across broad cross-pollination systems regardless of plant sex expression, provided that flowers exhibit variation in traits. Pollinators can evaluate this variation because to generate one seed via cross-pollination, a flower must assume a role as either female or male, which is known as the Fisher condition^39^.

To our knowledge, the sequential behavior of pollinators from male to female flowers has only been observed in obligate-mutualistic interactions, such as that between yucca plants and their pollinator, the yucca moth, and between Phyllanthaceae plants and their pollinators, *Epicephala* moths, where pollinators gain a clear benefit from sequential behavior, such as seeds for larval food^40,41^. However, the sequential behavior of pollinators that do not use seeds is equivocal in terms of the pollinator’s own benefit. A behavior without any obvious benefit might, however, still benefit the pollinators, because pollinators may learn to identify high-quality nectar. Indeed, pollinator behaviors based on their experience using memory or learning ability have been observed for species from a wide spectrum of taxa including flies, butterflies, and bees^42–52^.

Compared with pollinators that visit several co-flowering plants with different traits, the sequential behavior of pollinators visiting only one plant species may evolve readily, because they do not have the option to visit other co-flowering species. In future studies, we need to consider the community composition of flowering species and to conduct experiments using both naïve and affected pollinators that visit several plant species. The compositions of plant and pollinator community could change the likelihood or speed of the evolution of sexual dimorphisms in floral traits.

Furthermore, we, in future, need to consider more complicated and realistic conditions. In this study, we assumed a simple situation as the first step. For example, in the experience rule, the model supposed that pollinator can recognize floral traits accurately and behave based on their experience. Practically, some pollinators would sometimes recognize floral traits wrongly, and the probability of such false recognition would differ among pollinator species, sexes and individuals; the effects of false recognition on the evolution of floral traits remains to be explored. Another factor not included in our model is that the natural environment supports a diversity of plants and pollinators. If pollinators visit various flowering species, community composition of the flowering species would affect the nectar threshold that the pollinators will find favorably, because there will be inter-specific competition for pollinators as well as that within species. This means that we need to consider community composition as well as differences in pollinator species or individuals in simulation models.

Moreover, although our models assume one male flower can always give enough pollen for pollinators to pollinate female flowers, the number of flowers sufficient to provide pollen to pollinators might depend on the plant species and its pollinators. On *E. japonica* flowers, flies often visited several flowers before leaving male twigs, though we did not measure whether the number of flowers would affect the pollen amount on flies. In addition, we consider the setting as follows: when a pollinator visits a female flower after visiting a male flower, the female plant produces one seed, and all pollen is removed from the pollinator. However, how many visitations of pollinators are needed to set one seed or to drop all pollen would differ among plant species and their pollinators. Actually, a single visit of different pollinator species or individuals differently contribute to seed set, and a single visit may not give enough pollen to produce one seed^53,54^. If we change these simple assumptions, our simulation results would be changed.

In future, in order to test the sequence hypothesis more generally, we need to empirically measure a lot of parameters related with pollen movement and pollinator behavior, and then to construct simulations based on these complicated circumstances and parameters appropriate to actual systems. The future empirical and theoretical studies that we discussed above would tell us what kind of plant (e.g., dioecious, dichogamous hermaphrodite, etc.) or pollinator types would be well applied to the sequence hypothesis, or how often the sequence hypothesis can be applied in flowering plants.

In summary, we show that flowers with sexual dimorphism can benefit by invoking sequential behaviors from male flowers to female flowers in their pollinators. In the previously proposed evolutionary mechanisms, fitness benefit is considered separately for females and males. In this scenario, however, both female and male flowers gain equal fitness benefit.

## Materials and Methods

### Experiment

We used dioecious *E. japonica* plants that show sexually dimorphic floral traits in the site from where we collected twigs for experiment (Fig. 1). At this site, the seed set obtained with the artificial addition of pollen from the same population to female flowers was increased by 23% compared with that obtained with natural pollination (average seed set ± SE: 68.17 ± 0.21% with natural pollination and 91.33 ± 0.07% with artificial pollination), which suggests that pollinator attraction can determine both the female and male reproductive success rate of this plant^22^. The flowering season, floral longevity and flower number do not differ between sexes at the study site^32,55^ (see also Table S1 & S3). Female flowers have nectar with a significantly higher sugar concentration and a greater total sugar amount per flower (see Tables 1, S3 and S4). In contrast, male flowers have larger flowers (see Tables 1 & S3). Various fly, midge, bee, wasp, and beetle species visit flowers and remove nectar; the dominant flower visitors are Calliphoridae and Muscidae flies, and the flies carry pollen^22^. These insects will only visit *E. japonica* flowers in the absence of co-flowering species in early spring when *E. japonica* blooms^32^. This situation suggests that pollinators choose either male or female flowers of *E. japonica* and have no chance to experience other plant species. That is to say the flower visitors would have an effect on plant fitness as seasonal specialists at the study site, although they use other several plant species in different seasons as generalists.

*Eurya japonica* grows to a tree height of more than 10 m and a diameter at breast height (DBH) of 20 cm, and several hundred flowers develop on approximately 30-cm twigs (see also Table S2). The exact number of flowers per tree is unknown but almost reaches 0.1 million on a large tree^55^. Because this species is too large to observe the sequential foraging behavior of pollinators in the field, we conducted experiments in the laboratory using twigs. For this experiment, we used twigs with flowers collected from 10 female and 10 male plants from Kozagawa, Wakayama Prefecture, Japan (33°31′″50″N, 135°49′00″E, 30 m a.s.l.), where no spatial segregation in the locations of male and female trees is observed^30,32,55^. We used female and male individuals of two fly species that naturally visit *E. japonica* flowers, *Boettcherisca peregrina* (flesh fly, family: Sarcophagidae) and *Lucilia sericata* (green bottle fly, family: Calliphoridae), provided by Biotherapy Medical Corp. (Shiga, Japan) as pollinators. These pollinators were used one day after emergence and had thus never been exposed to flowers (i.e., naïve pollinators).

We conducted a two-choice experiment based on previous studies of pollinator behavior^56–58^ using a glass box (78 cm × 40 cm × 40 cm) with mesh windows on two sides placed under conditions consisting of a temperature 25 °C and 60% humidity (Fig. S3). To observe fly behavior, we placed female and male twigs in black bottles with water on each side of the box (see Fig. S3). The location of the two twigs was switched after each experimental run. We used twigs of similar size with similar numbers of flowers (i.e., about 30 cm twigs with about 300 flowers) as much as possible, and the twigs were renewed every day (Tables 1 & S2). Because we did not measure the sugar concentration in the nectar of the used flowers, we could not assess how the nectar quality differed from that of natural nectar in the field. One fly was removed from a test tube and placed at the center of the box. The behaviors of 340 naïve flies in total were observed with the 10 plant pairs in the daytime during the period of 1–30 March 2015 (Table S2). Preliminary observations in the daytime during 7–28 February 2015 revealed that when the flies visited female flowers, they spent less than 1 minute on a flower and visited several neighboring flowers for approximately 5 minutes. They then stopped nectar foraging and started the “bubbling behavior (defined by Gomes *et al*.^59^)” as follows. The flies regurgitated a droplet of fluid out and suck back in the buccopharyngeal cavity repeatedly before eventually ingesting. In contrast, when flesh flies visit male flowers (trees), after visiting one flower, they immediately move to a neighboring flower unless flying away. They never stays on the flower for an extended period of time. These flies walked around the twigs to visit many male flowers but immediately moving to another flowers. In the experiment, we waited until flies visited flowers naturally and recorded which twig the flies visited first, and we then waited for 1 minute and recorded whether the flies moved to the other twig. Using chi-squared test^23^, we examined whether the frequency of the fly movement form male flowers to female flowers differ from that of the movement from female flowers to male flowers.

### Model

A model focusing on flower size in a dioecious species was constructed. For successful pollination to occur in such plants, a pollinator needs to visit female flowers after visiting male flowers. If the pollinators visit flowers depending only on the flower size, the fitness of male and female individuals is expressed as

1 $$Wm=\frac{{s}_{m}^{\ast }}{{n}_{m}{s}_{m}+{n}_{f}{s}_{f}}\cdot \frac{{n}_{f}{s}_{f}}{{n}_{m}{s}_{m}+{n}_{f}{s}_{f}}$$

and

2 $${W}_{f}=\frac{{n}_{m}{s}_{m}}{{n}_{m}{s}_{m}+{n}_{f}{s}_{f}}\cdot \frac{{s}_{f}^{\ast }}{{n}_{m}{s}_{m}+{n}_{f}{s}_{f}}$$

respectively. Here, *n* and *s* indicate the number and size of flowers in male and female individuals (subscript) in the population, respectively. Asterisks indicate the flower size of a focal individual. To simplify the model, it was assumed that the pollinators visit only two flowers (i.e. one of four patterns: male and male flowers, male and female flowers, female and female flowers, female and male flowers), that a single visit on the male flower provide the pollinator with a sufficient amount of pollen, and that the pollen is transferred only to the next flower visited by the pollinator. These equations have a similar form for both sexes and the fitness of male and female individuals linearly increases with the flower size. Thus, the strength of the selection pressure on flower size will be similar between the sexes, and sexual dimorphism will not evolve under these conditions.

We subsequently consider the effect of reward in terms of the amount of nectar. If there is no nectar, pollinators will then visit flowers of different sizes. Even assuming this experience-dependent behavior of pollinators, naïve pollinators would choose large flowers. The effect of this experience can be written as follows:

3 $$E({s}_{1},{s}_{2})=1-\frac{1}{\exp (({s}_{1}-{s}_{2}{)}^{2})}$$

Here, *s*_1_ and *s*_2_ indicate the size of the last-visited and candidate flowers, respectively. This function reflects the negative impression of the size of a flower without nectar. A value of 0 is obtained if *s*_1_ = *s*_2_, whereas if *s*_2_ is notable different from *s*_1_, the function asymptotically approaches 1. Then, including this experience effect, the fitness of male and female individuals becomes

4 $$Wm=\frac{{s}_{m}^{\ast }}{{n}_{m}{s}_{m}+{n}_{f}{s}_{f}}\cdot \frac{E({s}_{m}^{\ast },{s}_{f}){n}_{f}{s}_{f}}{E({s}_{m}^{\ast },{s}_{m}){n}_{m}{s}_{m}+E({s}_{m}^{\ast },{s}_{f}){n}_{f}{s}_{f}}$$

and

5 $${W}_{f}=\frac{{n}_{m}{s}_{m}}{{n}_{m}{s}_{m}+{n}_{f}{s}_{f}}\cdot \frac{E({s}_{m},{s}_{f}^{\ast }){s}_{f}^{\ast }}{E({s}_{m},{s}_{m}){n}_{m}{s}_{m}+E({s}_{m},{s}_{f}){n}_{f}{s}_{f}}$$

respectively. This condition promotes the evolution of sexual dimorphism (*s*_*m*_ ≠ *s*_*f*_). These equations show that natural selection favors the conditions under which the functions *E*(*s*_*m*_, *s*_*m*_) and *E*(*s*_*m*_, *s*_*f*_) become small and large, respectively, which is achieved via sexual dimorphism. Therefore, sexual dimorphism would evolve under these conditions.

### Simulation

To test the above predictions, we constructed an individual-based model for an annual haploid dioecious species and its pollinators. The outline of the simulations is as follows. Each individual plant has two independent traits: flower size and quantity of nectar. Each trait is decided by a gene on a single sex-dependent locus. Thus, we assumed the occurrence of four loci for male and female flower size and male and female nectar volume. Pollinators visit flowers sequentially until they have visited 10 individual plants. If a pollinator visits a female flower after visiting a male flower, the female plant produces one seed, and all pollen is removed from the pollinator. If a pollinator visits a male flower after visiting a male flower, the pollen on the pollinator is replaced by that of the male visited later. In this simulation, it is assumed that a total of 1,000 pollinators perform this sequential pollination during the flowering season based on the three behavioral rules (detailed below). After pollination, all flowering individuals die, and the next generation begins with the seeds produced over the year. Before the beginning of the next generation, random mutations occur in the seeds at a constant probability per generation per locus (i.e., 0.001), and a random value from a standard normal distribution is then added to the genetic value.

We defined three rules that pollinators might use to decide which flowers to visit. The first rule is random choice, which assumes that pollinators randomly visit any flower among the entire population. The second is size-dependent choice, which assumes that pollinators favor larger flowers over other flowers. The probability that each fly would visit a particular flower was determined by the ratio of the flower size to the sum of all flower sizes, which is equal to that obtained with the conditions assumed by Eqs. 1 and 2. The third is choice depending on both size and experience, which assumes that naïve pollinators prefer to visiting large flowers, whereas experienced pollinators decide which flower to visit next based on their experience (the amount of nectar at the flower they visited last). If the amount is less than a certain threshold (set to 10 in this study), the pollinators disfavor the attribute of flower size when making decisions about flower visitation. The probability that the pollinators will visit a particular flower is the same as that obtained with the conditions assumed by Eqs. 4 and 5.

The simulations were initiated with 1,000 flowering individuals assigned random genetic values for both traits (a normal distribution: μ = 10, σ = 1) and were run for 10,000 generations. We performed 1,000 simulation runs for each pollinator rule. Because this simulation does not set any sexual difference as an initial value (male and female flowers have a similar size and a similar amount of nectar), whether sexual differences evolve depending on the pollinator’s behavior can be verified.

Of the total 1,000 simulation results, for each flower size and nectar amount, the numbers of simulation results where the female flower was larger than the male flower were statistically tested by the binomial test in R. The numbers of successful pollinations during the last generation of the simulations were also statistically compared among all three behavioral rules by ANOVA and Tukey HSD in R. Similarly, the numbers of successful pollinations within the results of experience rule were compared between the cases with larger male flowers and larger female flowers at the end of simulation by t test in R.

## Supplementary information

Supplementary information.

## Data Availability

The experimental data and the source code of the simulations written in C++ are shown in the supplementary information.
