# Supplementary material for: Dimorphic flowers modify the visitation order of pollinators from male to female flowers
Source: Sci Rep. 2020 Jun 19;10:9965. doi: 10.1038/s41598-020-66525-5 (PMC7305190; doi:10.1038/s41598-020-66525-5)
Supplement: Supplementary file 1 — Supplementary information. [file 41598_2020_66525_MOESM1_ESM.pdf]

Supplementary information

Dimorphic flowers modify the visitation order of pollinators from male to female flowers.

Kaoru Tsuji[1]†[*], Kazuya Kobayashi[2]†, Eisuke Hasegawa[3], Jin Yoshimura[4,5,6,7,8,9*]

[1] Center of Ecological Research, Kyoto University, Otsu, Shiga, 520-2113, Japan. http://orcid.org/0000-0001-5020-5184

[2] Hokkaido Forest Research Station, Field Science Education and Research Center, Kyoto University, 553 Tawa, Shibecha-cho, Kawakami-gun, Hokkaido 088-2339, Japan. http://orcid.org/0000-0002-9475-6807

[3] Laboratory of Animal Ecology, Department of Ecology and Systematics, Graduate School of Agriculture, Hokkaido University, Hokkaido 060-8589, Japan.

[4] Department of Mathematical and Systems Engineering, Shizuoka University, Hamamatsu, Japan.

http://orcid.org/0000-0003-1610-1386

[5] Department of International Health, Institute of Tropical Medicine Nagasaki University Nagasaki 852-8523 Japan

[6] Department of Environmental and Forest Biology, State University of New York College of Environmental Science and Forestry, Syracuse, New York, USA.

[7] Marine Biosystems Research Center, Chiba University, Kamogawa, Chiba, Japan.

[8] Department of Biological Sciences, Tokyo Metropolitan University, Hachioji, Tokyo, 192-0397 Japan.

[9] The University Museum, University of Tokyo, Bunkyo-ku, Tokyo, 113-0033 Japan.

† These authors equally contributed to this study

*Corresponding author: Kaoru Tsuji (tsuji@ecology.kyoto-u.ac.jp, Jin Yoshimura (yoshimura.jin@shizuoka.ac.jp)

1. Results

In the simulations with the random rule for pollinators, flower size (Fig. S1A) and nectar amount (Fig. S1C) randomly evolved in both sexes. The sexual differences in flower size (Fig. S1B) and nectar amount (Fig. S1D) are distributed around zero, and the number of simulation results with larger male flowers was 510 out of 1,000. In the size-dependent model, although large flower evolved (Fig. S2A), sexual dimorphism did not evolve in terms of flower size (Fig. S2B) or nectar amount (Fig. S2C,D), and the number of simulation results with larger male flowers was 460 out of 1,000.

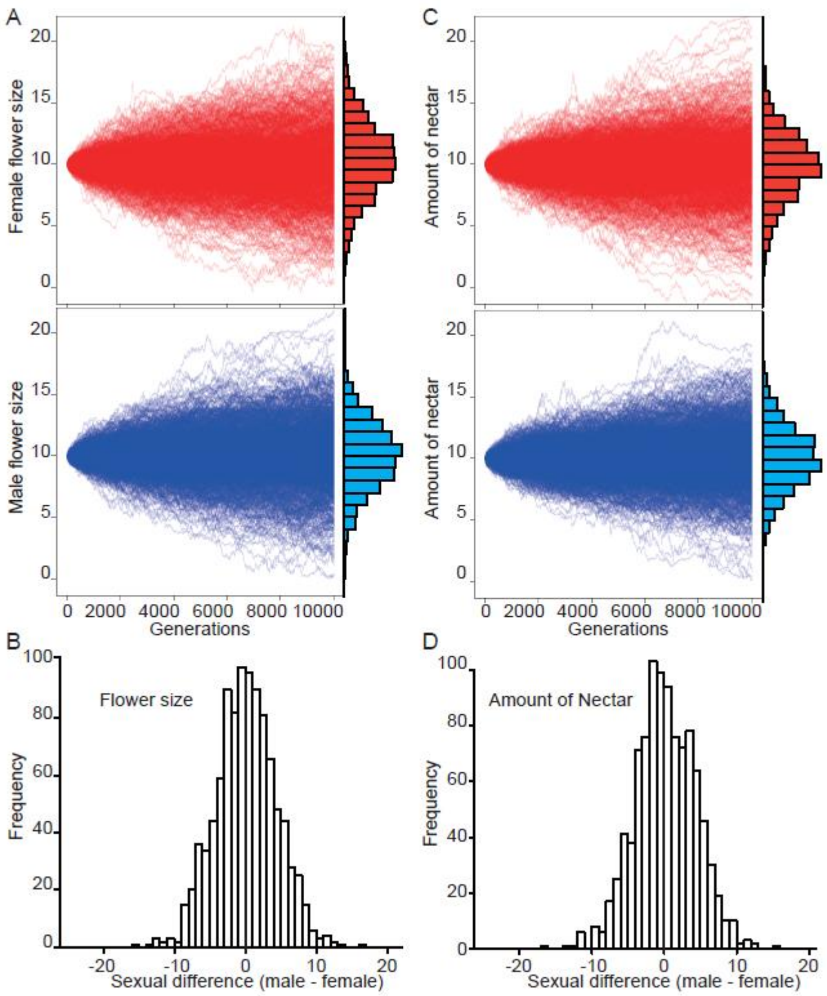

Figure S1. Simulation results for the pollinators using the random choice rule. (A) Evolutionary dynamics of female and male flower size, respectively. Each line represents a single simulation result. The distribution of flower size at the end of the simulations is shown as a histogram on the right side. (B) Sexual differences in flower size observed at the end of the simulations. (C) Evolutionary dynamics of amount of nectar in female and male flowers, respectively. Each line represents a single simulation result. The distribution of amounts of nectar at the end of the simulations is shown as a histogram on the right side. (D) Sexual differences in the amount of nectar observed at the end of simulations.

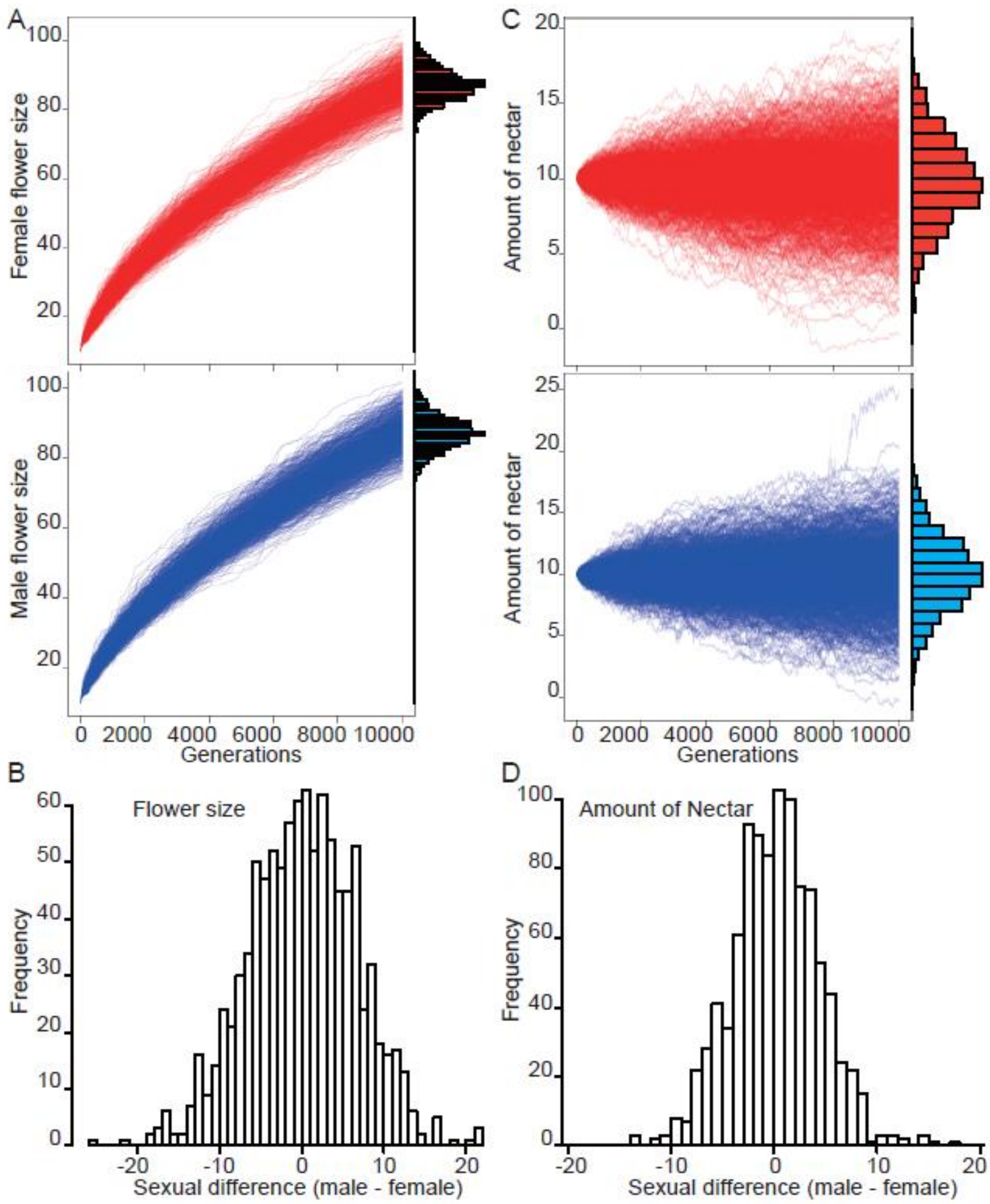

Figure S2. Simulation results for the pollinators using the size-dependent rule. (A) Evolutionary dynamics of female and male flower size, respectively. Each line represents a single simulation result. The distribution of flower size at the end of the simulations is shown as a histogram on the right side. (B) Sexual differences in flower size observed at the end of the simulations. (C) Evolutionary dynamics of amount of nectar for female and male flowers, respectively. Each line represents a single simulation result. The distribution of amounts of nectar at the end of the simulations is shown as a histogram on the right side. (D) Sexual differences in the amount of nectar observed at the end of the simulations.

Table S1. Analytical results for the effect of first or the other trials, and fly sex and species.

Proportion of on which flow flies visited was not significantly different between first trials and second and subsequent trails (χ^2 =3.85, P > 0.05).

	No. flies that visited female flowers	No. flies that visited male flowers	No. flies that visited female flowers and then moved to male flowers	No. flies that visited male flowers and then moved to female flowers
First trial	26	10	0	4
Second and subsequent trail	154	122	1	23

Effect of fly sex and species (* and ** show P<0.05 and <0.01, respectively)

	No. flies that visited female flowers	No. flies that visited male flowers	No. flies that visited female flowers and then moved to male flowers	No. flies that visited male flowers and then moved to female flowers
Flesh fly_female	48	45	0	6
Flesh fly_male	60	31*	0	10
Green bottle fly_female	33 **	40*	1	8
Green bottle fly_male	39 *	16	0	3

To analyze the frequency of fly movement, we used chi-squared test[1]. Although pollinator movement from male to female flowers was observed with two different fly species and with different fly sexes, the fly sex and species might affect their foraging. The movement in the one-minute observation was not cyclical. However, in nature, the movement may seem cyclical if pollinators might have short-term memory, do some other things after visiting female flowers (such as oviposition or searching partners or so), and forget favorable floral traits. As a consequence they would become the same as naïve pollinators and will visit a male flower again.

Table S2. Experimental design and detail results.

Plant pair ID	Day	Fly species_sex (no.)	No. female flowers	No. male flowers	Height of female twigs (cm)	Height of male twigs (cm)	No. flies that visited female flowers<br>Species _ sex (no.)	No. flies that visited male flowers<br>Species _ sex (no.)	No. flies that visited female flowers and then moved to male flowers<br>Species _ sex (no.)	No. flies that visited male flowers and then moved to female flowers<br>Species _ sex (no.)
1	3.1	F_M(1)	505	226	40	38	F_M(1)*	-	-	-
	3.2	F_M(2)	504	532	35	40	F_M(1)	-	-	F_M(1)*
	3.3	F_F(10),M(7)	421	609	25	30	F_F(7)*,M(4)	F_F(3),M(3)	-	-
	3.5	G_F(5),M(4)	478	419	40	35	G_F(2),M(3)	G_ F(3),M(1)*	-	-
	3.6	G_F(2),M(6)	538	529	25	30	G_M(3)	G_F(2),M(3)*	-	-
	3.7	G_F(3)	387	397	33	40	G_F(2)*	G_F (1)	-	-
2	3.4	F_F(3),M(7)	305	272	45	40	F_F(2)*,M(4)	F_M(2)	-	F_F(1),M(1)
	3.5	F_F(7),M(3)	221	212	55	30	F_F(6),M(3)*	F_F(1)	-	-
	3.7	G_F(1),M(2)	359	311	40	30	G_F(1),M(2)*	-	-	-
	3.8	G_F(4),M(2)	408	301	45	35	G_F(2),M(2)*	G_F(1)		G_F(1)
	3.9	G_F(1)	290	403	40	35	G_F(1)*	-	-	-
	3.10	G_M(2)	434	285	40	30	G_M(1)	G_M(1)*	-	-
	3.13	G_F(4),M(2)	229	302	30	30	G_F(2)	G_F(2)*,M(2)	-	-
	3.14	G_M(2)	348	229	35	30	G_M(2)*	-	-	-
3	3.7	F_F(10),M(10)	410	229	40	40	F_F(3)*,M(5)	F_F(7),M(5)	-	-
	3.13	G_F(3)	195	195	30	35	G_F(1)	G_F(1)	-	G_F(1)*
	3.15	G_F(7),M(2)	228	318	30	35	G_F(2)M(2)*	G_F(5)	-	-
	3.16	G_M(4)	266	310	35	35	G_M(3)*	G_M(1)	-	-
	3.20	G_M(4)	109	124	35	30	G_M(4)*	-	-	-

4	3.8	F_F(2),M(3)	352	262	30	30	F_F(2)*,M(1)	F_M(2)	-	-
	3.9	F_F(8),M(7)	366	356	30	35	F_F(3),M(4)*	F_F(5),M(1)	-	G_M(2)
	3.16	G_F(7)	238	215	30	40	G_F(2)*	G_F(4)	-	G_F(1)
	3.17	G_F(2)	200	199	30	35	G_F(1)*	G_F(1)	-	-
	3.19	G_F(1),M(2)	238	427	35	40	G_M(2)	G_F(1)*	-	
	3.21	G_M(8)	82	174	35	30	G_M(5)*	G_M(2)	-	G_M(1)
5	3.10	F_F(9),M(11)	515	333	40	35	F_F(3),M(6)	F_F(5)*,M(4)	-	F_F(1),M(1)
	3.24	G_F(10)	289	247	35	45	G_F(4)	G_F(5)*	-	G_F(1)
6	3.11	F_F(10),M(10)	280	228	40	40	F_F(4),M(7)*	F_F(6),M(2)	-	F_M(1)
	3.19	G_F(2)	340	379	40	30	-	G_F(1)	-	G_F(1)*
	3.20	G _F(8)	267	316	40	35	G_F(3)*	G_F(4)	-	G_F(1)
	3.22	G_M(10)	278	124	30	30	G_M(6)	G_M(3)*	-	G_M(1)
7	3.12	F_F(10),M(10)	315	254	30	35	F_F(3),M(6)	F_F(6),M(2)*	-	F_F(1),M(2)
8	3.14	F_F(10),M(10)	274	229	30	35	F_F(4),M(8)	F_F(5),M(1)	-	F_F(1)*,M(1)
9	3.17	F_F(10),M(9)	221	164	33	35	F_F(6)*,M(1)	F_F(4),M(8)	-	-
	3.19	F_M(1)	302	273	35	30	F_M(1)*	-	-	-
	3.23	F_F(10)	131	175	30	30	G_F(6)*	G_F(4)	-	-
10	3.25	F_F(9), M(5), G_F(7)	257	238	35	30	F_F(5),M(5) G_F(4)*	F_F(3), G_F(2)	-	F_F(1), G_F(1)
	3.26	F_F(1),M(5), G_F(3)	301	373	30	30	F_M(3)*	F_M(1) G_F(1)	G_F(1)	F_F(1),M(1) G_F(1)*
	3.27	G_M(3)	160	142	30	30	G_M(2)*	-	-	G_M(1)
	3.28	G_M(4)	235	203	30	30	G_M(2)	G_M(2)*	-	-
	3.30	G_F(2),M(1)	69	71	20	30	-	G_F(2),M(1)*	-	-

Fly species [F: flesh fly, G: green bottle fly], sex [F: female, M: male], * shows the number includes first trial in the day.

2. Materials and Methods

Table S3. Statistics

We used data published in Tsuji & Ohgushi (2018)[2], to analyze perianth length as the response variable with plant sex as fixed predictors and plant ID as a random effect. Furthermore, data published in Tsuji & Fukami (2018)[7] was used to analyze sugar amount per flower as the response variable with plant sex, bagging, and their interaction as fixed predictors and plant ID as a random effect. For these two statistical analyses, we used a generalized linear mixed model (GLMM) with a gamma distribution in the lme4 package[3] and the Type II Wald Chi-square test[4] in the car package[5] in R version 3.6.2[6]. Then, we analyze flower number shown in Table S2 as the response variable with plant sex, twig length, and their interaction as fixed predictors and plant ID as a random effect. We used a GLMM with a negative binomial distribution (lme4 package, and Type II Wald chi square tests in car package).

A. Perianth length

	χ^2	P
Sex	42.7	< 0.0001

B. Total sugar amount (μmol)

	χ^2	P
Bag	0.152	0.695
Sex	10.171	0.001
Bag*sex	6.957	0.008

C. Sucrose amount (μmol)

	χ^2	P
Bag	0.161	0.688
Sex	8.925	0.003
Bag*sex	3.945	0.047

D. Glucose amount (μmol)

	χ^2	P
Bag	0.057	0.811
Sex	8.597	0.003
Bag*sex	6.031	0.014

E. Fructose amount (μmol)

	χ^2	P
Bag	0.040	0.842

Sex	2.571	0.109
Bag*sex	4.331	0.037

F. Flower number

	χ^2	*P*
Twig length	2.6	0.1
Sex	0.3	0.6
Twig length*sex	0.2	0.7

Table S4. Sugar concentration and amount of nectar of bagged flowers

	Female plants (average ± SE)	male plants (average ± SE)
Total sugar amount per flower**	0.48 ± 0.03 μmol	0.11 ± 0.01μmol
Total sugar concentration*	0.08 ± 0.01 mol/L	0.07 ± 0.01 mol/L
Sucrose amount per flower**	0.36 ± 0.03 μmol	0.03 ± 0.00 μmol
Glucose amount per flower**	0.06 ± 0.00 μmol	0.01 ± 0.00 μmol
Fructose amount per flower	0.05 ± 0.00 μmol	0.01 ± 0.00 μmol
Sucrose concentration	0.08 ± 0.00 mol/L	0.07 ± 0.00 mol/L
Glucose concentration	0.02 ± 0.00 mol/L	0.01 ± 0.00 mol/L
Fructose concentration	0.01 ± 0.00 mol/L	0.02 ± 0.00 mol/L

We used data published in Tsuji & Fukami (2018)[7]. * and ** show <0.05 and 0.01, respectively.

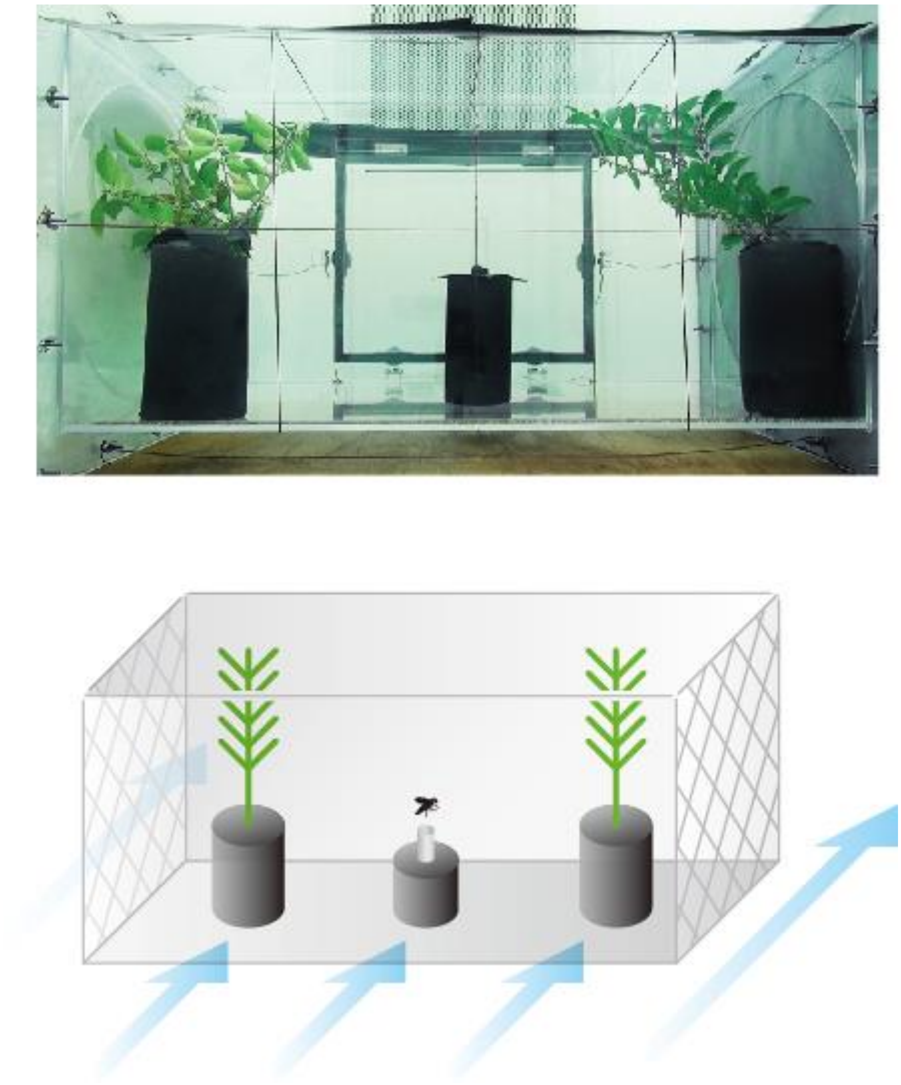

Figure S3. Image of the two-choice test. A test glass tube with a fly was set in the black holder at the center. Light blue arrows show air flow in the experimental room (25°C and 60% humidity, photograph: KT, illustration: YK). The humidity is similar to average humidity (62%) in March, flowering season of *E. japonica*[8]. The temperature we used was higher than average temperature (11°C) in March[9] because of logistical constraints, but the highest temperature in March was over 20°C[9], and 5 °C differences among near measurement places have been reported[10]. Furthermore, we recorded 25°C at our study site at 9 March 2020 when *E. japonica* set blooming flowers and flies moved actively and well visited flowers (see Figure S4).

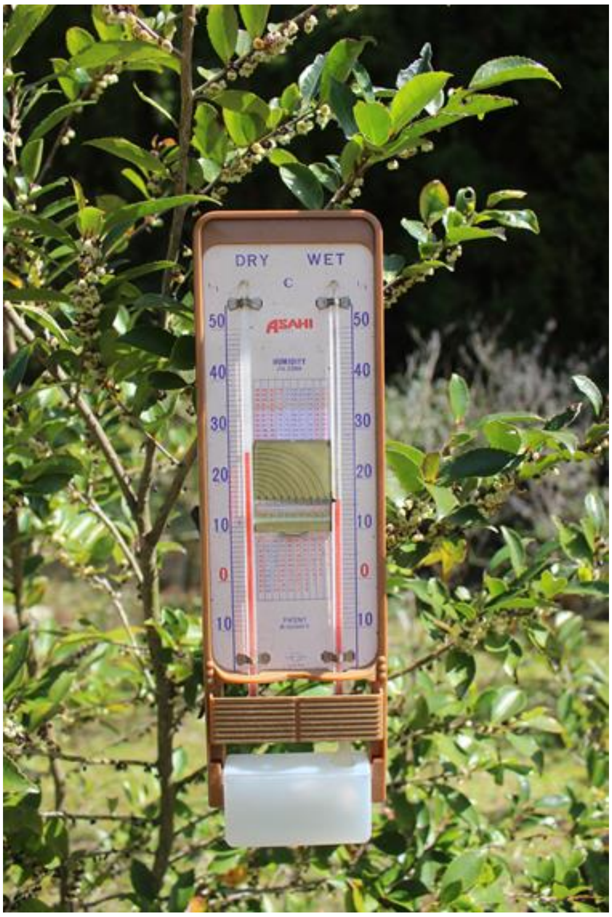

Figure S4. Photograph showing temperature (25.5°C, left red one) at the field site. This was taken at noon 9 March 2020, and behind tree was male *E. japonica* with blooming flowers.

Literature cited

1. http://www.kisnet.or.jp/nappa/software/star/freq/chisq_ixj.htm
2. Tsuji, K. & Ohgushi, T. Florivory indirectly decreases the plant reproductive output through changes in pollinator attraction. *Ecol. Evol.* **8,** 2993-3001 (2018).
3. Bates, D., Maechler, M., Bolker, B. & Walker, S. Fitting linear mixed-effects models using lme4. *J. Stat. Softw.* **67,** 1-48 (2015).
4. Langsrud, Ø. ANOVA for unbalanced data: Use Type II instead of Type III sums of squares. *Stat. Comput.* **13,** 163-167 (2003).
5. Fox, J. & Weisberg, S. An R Companion to Applied Regression, 2nd edition. Thousand Oaks California: Sage (2011).
6. R Core Team. R: A language and environment for statistical computing. R Foundation for Statistical Computing, Vienna, Austria. http://www.r-project.org/index.html (2019).
7. Tsuji, K. & Fukami, T. Community-wide consequences of sexual dimorphism: evidence from nectar microbes in dioecious plants. *Ecology* **99,** 2476-2484 (2018).
8. https://www.data.jma.go.jp/obd/stats/etrn/view/nml_sfc_ym.php?prec_no=65&block_no=47778&year=2015&month=3&day=&view=
9. https://www.data.jma.go.jp/obd/stats/etrn/view/daily_s1.php?prec_no=65&block_no=47778&year=2015&month=03&day=&view=p1
10. Tsuchiya, S., Kato, T., Tebakari, T. & Yamada, T. Mitigation effects of thermal environment of urban area by watering. *Proc. Hydraul. Eng.* **49** (in Japanese). https://www.pu-toyama.ac.jp/EE/tebakari/pdf/suikou49_uchimizu.pdf (2005).

Simulation code

```
// PlantSexDifference_v20180522.cpp

//#include "stdafx.h"
#include <random>
#include <iostream>
#include <fstream>
#include <sstream>
#include <string>
#include <time.h>
#include <vector>
#include <array>
#include <numeric>
#include <algorithm>
using namespace std;


int main(int argc,char *argv[])
{
	int i, j, k;

	//Read parameters from txt file
	double Parameters[10] = {};
	ifstream inputfile("Parameters.txt", ios::in); //read only
	string parameter_name;
	if (inputfile.fail()){
		cout << "Error: cannot open the text file (Parameters.txt)." << endl;
		return -1;
	}
	i = 0;
	while (true){//
		if (i % 2 == 0){
```

```
			inputfile >> parameter_name;
			if (inputfile.fail()) break;
			cout << parameter_name << " = ";
		}
		else {
			inputfile >> Parameters[i / 2];
			if (inputfile.fail()) break;
			cout << Parameters[i / 2] << endl;
		}
		i++;
	}
	inputfile.close();
	if(i != 22){
		cout << "Error: number of parameters is unexpected. i = " << i << endl;
		return -1;
	}
	cout << "parameter read end" << endl;

/* Example of "Parameters.txt"
NumInsects		1000
NumVisits		10
TimeSteps		10000
InitialPopulationSize	1000
InitialFemFlowerSize	10
InitialMalFlowerSize	10
InitialHoneydew		10
HoneydewThreshold	10
MutationRate		0.001
MutationSize		1.0
*/

	//Setting
```

```
int NumInsects = int(Parameters[0]);
int NumVisits = int(Parameters[1]);
int TimeSteps = int(Parameters[2]);
int InitialPopulationSize = int(Parameters[3]);
double InitialFemaleFlowerSize = Parameters[4];
double InitialMaleFlowerSize = Parameters[5];
double InitialNectar = Parameters[6];
double NectarThreshold = Parameters[7];
double MutationRate = Parameters[8];
double MutationSize = Parameters[9];

int MaxPopulationSize = NumInsects * (NumVisits - 1);

//allocate memory
vector<int> Sex(MaxPopulationSize, -1);                          //genetic value 1:female 0:male
vector<double> FemaleFlowerSize(MaxPopulationSize, -1);          //
vector<double> MaleFlowerSize(MaxPopulationSize, -1);        //
vector<double> FemaleNectar(MaxPopulationSize, -1);              //
vector<double> MaleNectar(MaxPopulationSize, -1);            //
vector<double> NextFemaleFlowerSize(MaxPopulationSize, -1);      //
vector<double> NextMaleFlowerSize(MaxPopulationSize, -1);    //
vector<double> NextFemaleNectar(MaxPopulationSize, -1);          //
vector<double> NextMaleNectar(MaxPopulationSize, -1);        //
vector<double> VisitingRate(MaxPopulationSize, -1);          //probability that naive insect visits the flower
vector<double> ExpVisitingRate(MaxPopulationSize, -1);       //probability that experienced insect visits the flower

//allocate memory for output
vector<double> MeanFemaleFlowerSizeOutput(TimeSteps, 0);
vector<double> MeanMaleFlowerSizeOutput(TimeSteps, 0);
vector<double> MeanFemaleNectarOutput(TimeSteps, 0);
vector<double> MeanMaleNectarOutput(TimeSteps, 0);
vector<int> PopulationSize(TimeSteps, 0);
```

```
//Random function
random_device rd;      // non-deterministic generator
mt19937 gen(rd());    // to seed mersenne twister.
uniform_int_distribution<int> rbinom(0, 1);                                          //random binomial
uniform_real_distribution<double> runif(0.0, 1.0);                                   //random uniform
normal_distribution<double> rnorm(0.0, MutationSize);                                //random normal


cout << "simulation start" << endl;
//initialize population
int CurrentPopulationSize = InitialPopulationSize;
for (i = 0; i < InitialPopulationSize; i++){
	Sex[i] = rbinom(gen);
	FemaleFlowerSize[i] = InitialFemaleFlowerSize + rnorm(gen);
	MaleFlowerSize[i] = InitialMaleFlowerSize + rnorm(gen);
	FemaleNectar[i] = InitialNectar + rnorm(gen);
	MaleNectar[i] = InitialNectar + rnorm(gen);
}


for (int t = 0; t < TimeSteps; t++){
	//Calculate the effect of flower size on the probability that pollinators visit
	VisitingRate[0] = FemaleFlowerSize[0] * Sex[0] + MaleFlowerSize[0] * (1 - Sex[0]);
	for (i = 1; i < CurrentPopulationSize; i++){
		VisitingRate[i] = VisitingRate[i-1] + FemaleFlowerSize[i] * Sex[i] + MaleFlowerSize[i] * (1 - Sex[i]);
	}

	//reset
	int VisitingFlowerID = -1;
	int VisitingFlowerSex = -1;
	int PastVisitingFlowerID = -1;       //store pollen parent id
```

```
int n = 0;                                      //count produced seeds
double Experience = 0.0;
double Threshold, randomNum, TheFlowerSize, ExperiencedFlowerSize;

for (i = 0; i < NumInsects; i++){
	VisitingFlowerID = -1;
	VisitingFlowerSex = -1;
	PastVisitingFlowerID = -1; //reset

/*completely random choice*/
	for (j = 0; j < NumVisits; j++){
		VisitingFlowerID = int(CurrentPopulationSize * runif(gen));
		VisitingFlowerSex = Sex[VisitingFlowerID];
		if (VisitingFlowerSex){ //if the flower is female
			if (PastVisitingFlowerID != -1){ //if the insect has pollen
				//Polination success, seed production
				if (rbinom(gen)){
					NextFemaleFlowerSize[n] = FemaleFlowerSize[VisitingFlowerID];    //inherit from mother
				}
				else{
					NextFemaleFlowerSize[n] = FemaleFlowerSize[PastVisitingFlowerID];     //inherit from father
				}
				if (rbinom(gen)){
					NextMaleFlowerSize[n] = MaleFlowerSize[VisitingFlowerID];   //inherit from mother
				}
				else{
					NextMaleFlowerSize[n] = MaleFlowerSize[PastVisitingFlowerID];    //inherit from father
				}
				if (rbinom(gen)){
					NextFemaleNectar[n] = FemaleNectar[VisitingFlowerID];   //inherit from mother
				}
				else{
```

```
                    NextFemaleNectar[n] = FemaleNectar[PastVisitingFlowerID];    //inherit from father
                }
                if (rbinom(gen)){
                    NextMaleNectar[n] = MaleNectar[VisitingFlowerID]; //inherit from mother
                }
                else{
                    NextMaleNectar[n] = MaleNectar[PastVisitingFlowerID];   //inherit from father
                }
                n++;
                PastVisitingFlowerID = -1;    //remove the pollen on the insect
            }
        }
        else {//if it is male
            PastVisitingFlowerID = VisitingFlowerID;    //replace the pollen on the insect
        }
    }
/*completely random choice end*/



/*experience-dependent choice
    //Naive insects choose a flower by the size
    randomNum = runif(gen);
    Threshold = VisitingRate[CurrentPopulationSize - 1] * randomNum;
    int expected_VisitingFlowerID = int(randomNum * CurrentPopulationSize);
    if (Threshold > VisitingRate[expected_VisitingFlowerID]){
        for (j = expected_VisitingFlowerID + 1; j < CurrentPopulationSize; j++){
            if (Threshold < VisitingRate[j]){
                break;
            }
        }
    }
```

```
else{
        for (j = expected_VisitingFlowerID; j >= 0; j--){
                if (Threshold >= VisitingRate[j]){
                        j++;
                        break;
                }
        }
}
if (j < 0){ j = 0; }
VisitingFlowerID = j;
VisitingFlowerSex = Sex[VisitingFlowerID];
if (VisitingFlowerSex){ //if the flower is female
        if (FemaleNectar[VisitingFlowerID] > NectarThreshold){
                //Experience = 1.0;           //it has a positive effect
                Experience = 0.0;             //it has no effect
        }
        else{
                //Experience = -1.0;          //it has a negative effect
                Experience = 0.0;             //it has no effect
        }
        ExperiencedFlowerSize = FemaleFlowerSize[VisitingFlowerID];
        PastVisitingFlowerID = -1;
}
else {//if it is male
        if (MaleNectar[VisitingFlowerID] > NectarThreshold){
                //Experience = 1.0;           //it has a positive effect
                Experience = 0.0;             //it has no effect
        }
        else{
                //Experience = -1.0;          //it has a negative effect
                Experience = 0.0;             //it has no effect
        }
```

```
        ExperiencedFlowerSize = MaleFlowerSize[VisitingFlowerID];
        PastVisitingFlowerID = VisitingFlowerID;
    }

    for (j = 1; j < NumVisits; j++){
        //Add the effect of experience
        for (k = 0; k < CurrentPopulationSize; k++){
            TheFlowerSize = FemaleFlowerSize[k] * Sex[k] + MaleFlowerSize[k] * (1 - Sex[k]);
            double SizeDifference = TheFlowerSize - ExperiencedFlowerSize;
            ExpVisitingRate[k] = VisitingRate[k] + (Experience * TheFlowerSize) / exp(SizeDifference * SizeDifference);
        }
        randomNum = runif(gen);
        Threshold = ExpVisitingRate[CurrentPopulationSize - 1] * randomNum;
        int expected_VisitingFlowerID = int(randomNum * CurrentPopulationSize);
        if (Threshold > ExpVisitingRate[expected_VisitingFlowerID]){
            for (k = expected_VisitingFlowerID + 1; k < CurrentPopulationSize; k++){
                if (Threshold < ExpVisitingRate[k]){
                    break;
                }
            }
        }
        else{
            for (k = expected_VisitingFlowerID; k >= 0; k--){
                if (Threshold >= ExpVisitingRate[k]){
                    k++;
                    break;
                }
            }
        }
        if (k < 0){ k = 0; }
        VisitingFlowerID = k;
        VisitingFlowerSex = Sex[VisitingFlowerID];
```

```
if (VisitingFlowerSex){ //if the flower is female
	if (FemaleNectar[VisitingFlowerID] > NectarThreshold){
		Experience = 1.0;		//it has a positive effect
		//Experience = 0.0;		//it has no effect
	}
	else{
		Experience = -1.0;		//it has a negative effect
		//Experience = 0.0;		//it has no effect
	}
	ExperiencedFlowerSize = FemaleFlowerSize[VisitingFlowerID];
	if (PastVisitingFlowerID != -1){ //if the insect has pollen
		//Polination success, seed production
		if (rbinom(gen)){
			NextFemaleFlowerSize[n] = FemaleFlowerSize[VisitingFlowerID];	//inherit from mother
		}
		else{
			NextFemaleFlowerSize[n] = FemaleFlowerSize[PastVisitingFlowerID];	//inherit from father
		}
		if (rbinom(gen)){
			NextMaleFlowerSize[n] = MaleFlowerSize[VisitingFlowerID];	//inherit from mother
		}
		else{
			NextMaleFlowerSize[n] = MaleFlowerSize[PastVisitingFlowerID];	//inherit from father
		}
		if (rbinom(gen)){
			NextFemaleNectar[n] = FemaleNectar[VisitingFlowerID];	//inherit from mother
		}
		else{
			NextFemaleNectar[n] = FemaleNectar[PastVisitingFlowerID];	//inherit from father
		}
		if (rbinom(gen)){
			NextMaleNectar[n] = MaleNectar[VisitingFlowerID]; //inherit from mother
```

```
                }
                else{
                    NextMaleNectar[n] = MaleNectar[PastVisitingFlowerID];   //inherit from father
                }
                n++;
                PastVisitingFlowerID = -1;    //remove the pollen on the insect
            }
        }
        else {//if it is male
            if (MaleNectar[VisitingFlowerID] > NectarThreshold){
                Experience = 1.0;                  //it has a positive effect
                //Experience = 0.0;                //it has no effect
            }
            else{
                Experience = -1.0;                 //it has a negative effect
                //Experience = 0.0;                //it has no effect
            }
            ExperiencedFlowerSize = MaleFlowerSize[VisitingFlowerID];
            PastVisitingFlowerID = VisitingFlowerID;                        //replace the pollen on the insect
        }
    }
    /*experience-dependent choice end*/

}//Pollination end (repeat for each insect)


//mutation & replace
for (i = 0; i < n; i++){
    if (runif(gen) < MutationRate){
        NextFemaleFlowerSize[i] += rnorm(gen);
    }
    if (runif(gen) < MutationRate){
```

```
            NextMaleFlowerSize[i] += rnorm(gen);
        }
        if (runif(gen) < MutationRate){
            NextFemaleNectar[i] += rnorm(gen);
        }
        if (runif(gen) < MutationRate){
            NextMaleNectar[i] += rnorm(gen);
        }
        FemaleFlowerSize[i] = NextFemaleFlowerSize[i];
        MaleFlowerSize[i] = NextMaleFlowerSize[i];
        FemaleNectar[i] = NextFemaleNectar[i];
        MaleNectar[i] = NextMaleNectar[i];
        Sex[i] = rbinom(gen);

        //for output
        MeanFemaleFlowerSizeOutput[t] += FemaleFlowerSize[i];
        MeanMaleFlowerSizeOutput[t] += MaleFlowerSize[i];
        MeanFemaleNectarOutput[t] += FemaleNectar[i];
        MeanMaleNectarOutput[t] += MaleNectar[i];
    }
    CurrentPopulationSize = n;
    PopulationSize[t] = CurrentPopulationSize;

    //for output of flowerSize at the timestep
    MeanFemaleFlowerSizeOutput[t] /= CurrentPopulationSize;
    MeanMaleFlowerSizeOutput[t] /= CurrentPopulationSize;
    MeanFemaleNectarOutput[t] /= CurrentPopulationSize;
    MeanMaleNectarOutput[t] /= CurrentPopulationSize;
}//end of timestep

//output
time_t t = time(NULL);
```

```
const tm* lt = localtime(&t);
char str[81];
strftime(str,sizeof(str),"%Y%m%d_%H%I%S", lt);

ostringstream FP1;
ostringstream FP2;
ostringstream FP3;
ostringstream FP4;
ostringstream FP5;
FP1 <<str<<"Rep"<<argv[1]<<"NI"<<NumInsects<<"NV"<<NumVisits<<"IFS"<<InitialFemaleFlowerSize<<"MR"<<MutationRate<<"FemaleFS.csv";
FP2 <<str<<"Rep"<<argv[1]<<"NI"<<NumInsects<<"NV"<<NumVisits<<"IFS"<<InitialFemaleFlowerSize<<"MR"<<MutationRate<<"MaleFS.csv";
FP3 <<str<<"Rep"<<argv[1]<<"NI"<<NumInsects<<"NV"<<NumVisits<<"IFS"<<InitialFemaleFlowerSize<<"MR"<<MutationRate<<"FemaleAN.csv";
FP4 <<str<<"Rep"<<argv[1]<<"NI"<<NumInsects<<"NV"<<NumVisits<<"IFS"<<InitialFemaleFlowerSize<<"MR"<<MutationRate<<"MaleAN.csv";
FP5 <<str<<"Rep"<<argv[1]<<"NI"<<NumInsects<<"NV"<<NumVisits<<"IFS"<<InitialFemaleFlowerSize<<"MR"<<MutationRate<<"PopSize.csv";
ofstream ofs1(FP1.str());
ofstream ofs2(FP2.str());
ofstream ofs3(FP3.str());
ofstream ofs4(FP4.str());
ofstream ofs5(FP5.str());
for (j = 0; j < TimeSteps; j++){
	ofs1 << MeanFemaleFlowerSizeOutput[j] << ",";
	ofs2 << MeanMaleFlowerSizeOutput[j] << ",";
	ofs3 << MeanFemaleNectarOutput[j] << ",";
	ofs4 << MeanMaleNectarOutput[j] << ",";
	ofs5 << PopulationSize[j] << ",";
}
ofs1 << endl;
ofs2 << endl;
ofs3 << endl;
ofs4 << endl;
ofs5 << endl;
```

```
    cout << "Simulation finished!" << endl;
    return 0;

}
```
